# Efficacy of *Momordica charantia* in glycaemic control and insulin resistance among patients with prediabetes and type 2 diabetes. A GRADE-adherent meta-analysis of randomised controlled trials

**DOI:** 10.1016/j.metop.2025.100407

**Published:** 2025-10-24

**Authors:** Sphesihle A.L. Mkhize, Wendy N. Phoswa, Phikelelani S. Ngubane, Kabelo Mokgalaboni

**Affiliations:** a Department of Life and Consumer Sciences, College of Agriculture and Environmental Sciences, University of South Africa, Florida Campus, Roodepoort, 1709, South Africa; b Department of Physiology, School of Laboratory Medicine and Medical Sciences, University of KwaZulu-Natal, Westville Campus, Durban, 4001, South Africa

**Keywords:** Hyperglycaemia, *Momordica charantia*, Bitter melon, Prediabetes, Type 2 diabetes

## Abstract

The prevalence of prediabetes is rising globally, and if untreated, it can lead to a surge in the rate of type 2 diabetes (T2D). Altogether, these conditions are characterised by hyperglycaemia, which promotes cardiovascular complications. Although Momordica has shown promising results in preclinical studies, the existing quantitative synthesis of evidence reports contradictory findings. This study aimed to evaluate the effect of *Momordica charantia* on fasting blood glucose (FBG), glycated haemoglobin (HbA1c), insulin, homeostatic model of insulin resistance (HOMA-IR), and homeostatic model of β-cell function (HOMA-β) in individuals with prediabetes or T2D. A search for randomised controlled trials was conducted on Scopus, PubMed, and Web of Science until July 19, 2025. Keywords used included *M. charantia*, bitter melon, prediabetes and diabetes mellitus. The data were presented as standard mean difference (SMD) and 95 % confidence intervals. Twenty-five trials with 34 sub-studies were deemed relevant. The results showed reduced FBG [SMD = −0.46 (−0.73, - 0.18), *p* = 0.0012] and HbA1c [SMD = −0.57 (−0.83, −0.31), *p* < 0.0001]. Additionally, there was a significant reduction in the level of insulin [SMD = −0.48 (−0.83, −0.12), *p* = 0.0082] and HOMA-IR [SMD = −0.52 (−0.95, −0.08), *p* = 0.0195]. However, no effect was observed on HOMA-β (*p* = 0.586). The study findings suggest that *M. charantia* may be used to improve insulin sensitivity and reduce insulin resistance, thereby improving hyperglycaemia in patients with prediabetes and T2D.

## 1 Introduction

The prevalence of cardiometabolic diseases is globally rising, and this increases the risk of cardiovascular diseases and associated mortality [1]. Cardiometabolic diseases refer to a group of conditions marked by the concurrence of risk factors, including obesity, hyperglycaemia, dysregulated endocrine functions, and insulin resistance [2]. Insulin resistance may progress into prediabetes, and subsequently into type 2 diabetes mellitus (T2D) when left unmanaged [3,4]. These conditions are marked by high blood glucose; however, prediabetes is marked by high glucose levels, but not high enough to be classified as T2D [5]. According to the American Diabetes Association, prediabetes is defined as fasting blood glucose (FBG) levels ranging between 100 and 125 mg/dL or glycated haemoglobin (HbA1c) of 5.7–6.4 %, whereas in diabetes (DM), FBG and HbA1c are greater than 126 mg/dL and 6.5 % respectively [6]. It is worth noting that the prevalence of prediabetes was reportedly high in 2021, and this is anticipated to rise [7]. This suggests that the risk of T2D is also anticipated to rise, provided the prediabetes is not adequately controlled. According to the International Diabetes Federation (IDF), it is estimated that globally, approximately 1 in 9 adults has DM [8]. This suggests that the CVD associated with T2D may be higher globally. During the progression of prediabetes to T2D, the antioxidant defence mechanisms of the body are altered, resulting in a buildup of reactive oxygen species (ROS), which can promote secondary complications [9,10]. The hyperglycaemic states promote oxidative stress by increasing the production of ROS, and this induces lipid peroxidation, which damages the beta cells, resulting in apoptosis, subsequently in decreased insulin secretion [[11], [12], [13]]. Hyperglycaemia can damage various tissues, including the retina, blood vessels, and renal function [14,15]. Therefore, managing hyperglycaemic status in prediabetes is important to curb its progression into DM and its associated complications. Several antihyperglycaemic treatments have been reported; however, their cost, side effects and related complications promote reluctance and defaulting [[16], [17], [18]]. This prompts the need for an alternative approach to control hyperglycaemia in both prediabetes and T2D.

Existing evidence shows that humans have been using plants and herbs to treat various chronic conditions and diseases, or to alleviate their symptoms [19]. One notable example is *Momordica charantia* (*M. charantia*), commonly known as bitter melon [20,21]. This plant belongs to the family Cucurbitaceae and is native to tropical regions, including Asia and some parts of Africa [20]. *M. charantia* has phytochemicals that include proteins and peptides, phenolics, lipids, terpenoids, and saponins [[22], [23], [24]]. These compounds exhibit hypoglycaemic effects in animals that mimic human prediabetic and DM status, as reported elsewhere [25,26]. However, the translatability of the above evidence in clinical settings is not properly validated. For instance, in overweight males, *M. charantia* showed no effect on FBG [27]. This same observation is also noted in those with T2D [28,29]. Recently, a quantitative study on *M. charantia* confirmed no effect on FBG [30]. Although another meta-analysis confirmed the hypoglycaemic effect of *M. charantia* in T2D, the evidence was derived from only eight trials, which might limit the overall statistical power [31]. These observations suggest that there are limitations in the translatability of preclinical studies into clinical settings. Therefore, this study aimed to systematically assess the efficacy of *M. charantia* compared with placebo or active controls on FBG, HbA1c, insulin, homeostatic model of insulin resistance (HOMA-IR) and HOMA-β in adults with prediabetes or type 2 diabetes. The assessment followed the grading of recommendations, assessment, development, and evaluation (GRADE) framework to ensure transparency, methodological rigor and high-quality evidence.

## 2 Method

### 2.1 Protocol registration and PICOS criteria

This study is a systematic review and meta-analysis, registered with PROSPERO under registration code: CRD420251086536. It followed the updated Preferred Reporting Items for Systematic Review and Meta-Analysis (PRISMA) and adhered to the PICOS guidelines [32,33]. The PICOS criteria as stipulated in the registered protocol were defined as follows: individuals living with prediabetes or T2D, the intervention was *M. charantia* therapy (single or combined therapy), a comparator was placebo or hypoglycaemic drug, the outcome included FBG, HbA1c, insulin, HOMA-IR and HOMA-β and study design as randomised controlled trials (RCTs). This study is reported in accordance with the PRISMA checklist (Appendix A).

### 2.2 Information source and search strategy

PubMed, Scopus, Web of Science and manual screening were used to conduct a comprehensive literature search. The following Medical Subject Headings (MeSH) terms and Boolean operators: “diabetes mellitus” OR “prediabetes”, AND “*Momordica charantia”* OR “bitter melon”. The search terms were adapted for each database, without restriction on publication dates; therefore, the results included studies published from the inception of the database until the 19th of July 2025. The filters used included design (randomised controlled trials), language (English), and publication types (mainly journal articles), primarily sourced from Scopus. The search was done independently by (SALM and KM) to avoid any bias. In the event of disagreement, a third independent researcher (WNP) used the same key and MeSH terms to verify the search results.

### 2.3 Eligibility criteria and selection process

The studies adhere to the following criteria: participants with prediabetes or T2D, *M. charantia* extract and powder, a control group (placebo or any hypoglycaemic agent), and outcomes included FBG, insulin, HbA1c, HOMA-IR and HOMA-β. The following studies were excluded: those conducted in languages other than English, rodent and cell culture studies, and those addressing other conditions. Only full-text results were included in this review. The screening was conducted by independent researchers (SALM and KM) to minimise any potential bias. In the event of disagreement, two independent researchers (WNP and PSN) used the preplanned PICOS to verify the selected trials.

### 2.4 Data items and extraction

The data extracted by two independent researchers (SALM and KM) from each study, included the surname of the first author, the year that the study was published, the country where the study was conducted, the study design, and the intervention information (the part of *M. charantia,* dose, duration of intervention), number of males and female in each study, body mass index (BMI), and the main results of the study (FBG, HbA1c, insulin, HOMA-IR and HOMA-β). The trials with multiple treatment arms (higher and lower doses) were treated as different sub-studies. For any disagreement in extraction between (SALM and KM), a third independent researcher (WNP) took over by assessing the trial, items and data in question.

### 2.5 Quality, risk of bias, and certainty of evidence

The quality of each trial was independently assessed by using the revised Cochrane risk of bias tool (ROB) and generated using the ROB visualisation (robvis) web tool [34,35]. This tool considers five main domains, which include (D1) bias due to randomisation, (D2) bias due to deviation from the intended intervention, (D3) bias due to missing data, (D4) bias due to outcome assessment, and (D5) bias due to the selection of reported results. The overall quality of individual trials was classified based on the level of bias across different domains. The trial was considered to have good or low quality based on whether it had a low or high risk of bias, respectively. A trial was rated as moderate quality if it was rated with some concerns. The trial was classified as low risk (high quality) if it was judged to have a low risk of bias in all five domains. Additionally, the study was considered to have some concerns (moderate quality) if one domain had concerns; it had a low risk in four domains, without any high-risk domains. Lastly, a study that had at least one domain judged as high risk was ultimately rated as high risk (low quality). Certainty of evidence was evaluated by adopting the grading of recommendations, assessment, development, and evaluation (GRADE) approach [36]. This approach also considers five domains, including (1) risk of bias, (2) inconsistency, (3) indirectness, (4) imprecision and (5) publication bias. The evidence was upgraded based on the large effect size, plausible confounding, or dose-dependent relationship. The overall certainty was classified as either low, very low, moderate, or good evidence, and presented as a summary of the findings table generated through GRADEpro software, https://www.gradepro.org/(accessed July 22, 2025).

### 2.6 Data synthesis and analysis

From each trial, the sample size, mean, and standard deviation from each group were extracted and computed into the meta-analysis web tool [37]. In case the study did not report the change in the mean and SD, the mean change was determined as the difference between post-treatment and pretreatment using the formula, change in SD = square root of [(SD_b_)^2^ +(SD_f_)^2^ –2R(SD_b_ x SD_f_)], with correlation (R) of 0.5 as previously used [38]. If the study reported median and interquartile range (IQR), we estimated the mean and SD using the procedure described by Abbas et al., 2024 [39]. The data from the male and female groups were combined into a single dataset using the Cochrane formula, https://www.statstodo.com/CombineMeansSDs.php (accessed on July 07, 2025). The heterogeneity was quantified using the *I*^2^ statistical test [40]. The *I*^2^ values below 0 % indicated a lack of heterogeneity, while those of 50 % or greater were classified as substantial statistical heterogeneity. We used a fixed-effects model in the absence of statistical heterogeneity and a random-effects model in its presence [41]. We conducted a subgroup analysis to identify the sources of heterogeneity, based on the dose of *M. Charantia*, duration of intervention, and ROB. Publication bias was assessed through visual inspection of funnel plots and statistically through Egger's test [42]. The trim and fill tests were also used to adjust for publication bias. Sensitivity analysis was conducted using one study exclusion approach and reanalysis of the effect size to confirm the stability of the results [43]. The results were presented as a forest plot and funnel plots. The statistical significance threshold was set at *p* < 0.05 for all statistical tests.

## 3 Results

### 3.1 Literature search and selection procedure

**Fig. 1:**
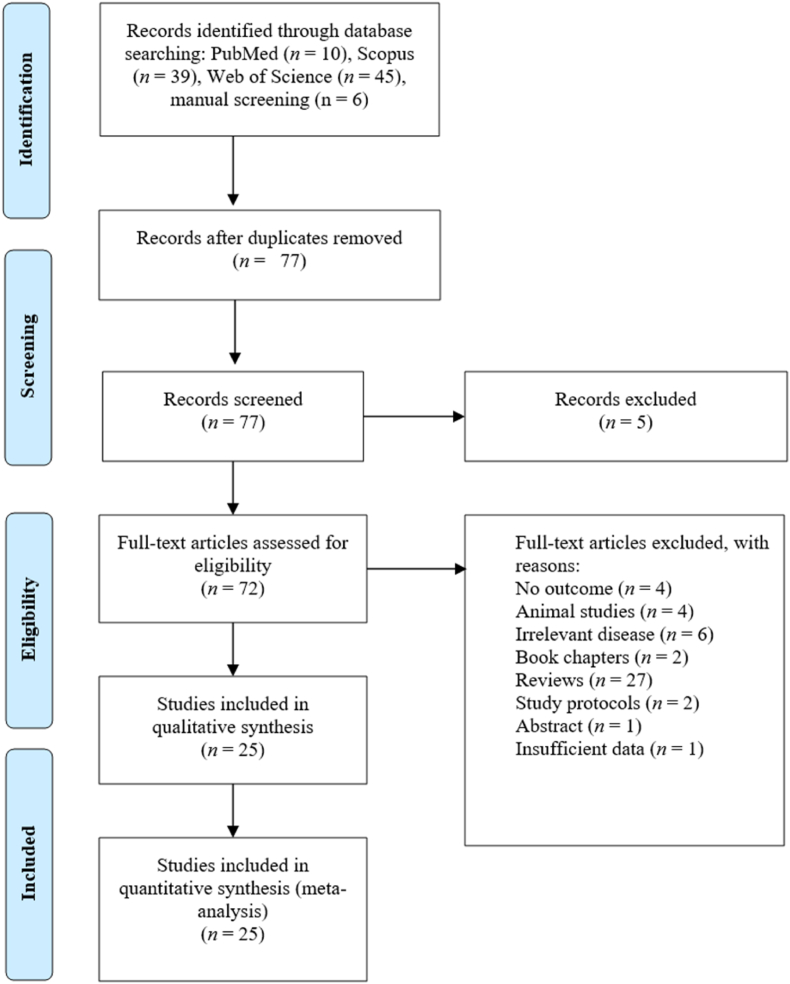
A PRISMA flow diagram showing the literature collection and screening procedure.

Ninety-four records were identified from three online databases: ten from PubMed, 39 from Scopus, and 45 from the Web of Science (Appendix B Table 1). Additionally, six more were obtained through a manual screening of relevant references. Before screening, all records were combined in a Microsoft Excel sheet (version 2503). After filtering the records in ascending alphabetical order, 23 records were identified as duplicates and thus excluded. Following the initial phase of screening the records, which involved title, abstract, and keywords, an additional five records were excluded because their titles were irrelevant. Of those that remained, their full texts were sought, retrieved and read in full. However, the remaining records were deemed irrelevant for the following reasons: four did not report any of the outcomes, and six involved participants with conditions other than prediabetes or T2D (healthy, osteoarthritis, metabolic syndrome, and obesity). Additional reasons included two study protocols, four rodent studies (involving fish, mice, and rats), one conference abstract, one report of baseline data without accompanying post-intervention data, two book chapters, and 27 review articles. Of the outstanding 25 records, one full text was not publicly available; however, the main author was contacted to share it [44]. Therefore, only 25 trials [28,29,[44], [45], [46], [47], [48], [49], [50], [51], [52], [53], [54], [55], [56], [57], [58], [59], [60], [61], [62], [63], [64], [65], [66]] with enough data were included in the final analysis (Fig. 1).

### 3.2 Characteristics of included studies

The study included 25 trials with 34 substudies [28,29,[44], [45], [46], [47], [48], [49], [50], [51], [52], [53], [54], [55], [56], [57], [58], [59], [60], [61], [62], [63], [64], [65], [66]] published between 2003 and 2025 in peer-reviewed journals, with the majority in 2018. Among them, 24 studies were conducted in individuals with T2D, and 11 in those with prediabetes. The published studies were conducted in a wide number of countries, including Pakistan, the Netherlands, the USA, Thailand, Korea, Taiwan, Mexico, Tanzania, India, Germany, China and the Philippines. Other studies employed a combination therapy or polyherbal formulation of *M. charantia* powdered capsules with other herbs [45,46,53,[61], [62], [63], [64]], micronutrients [57], while others used bitter melon as juice or a single powdered capsule [28,29,44,[47], [48], [49], [50], [51], [52],[54], [55], [56], [57], [58], [59], [60],^63^,^65^,^66^]. In one trial [53], insulin resistance was assessed using the HOMA-IR calculation. The overall demographic overview of the included studies is presented in Table 1.

**Table 1:** Characteristics of included trials.

Author, year	Country	Study design,	Condition and sample	Grouping Final sample	Intervention	Form of drug	Mean age ± SD (intervention vs control)	Mean BMI±SD (intervention vs control)	Gender (M/F)	Glycaemic markers reported
Khalid et al., 2025 [65]	Pakistan	Randomised controlled trial	60 T2D	30 on bitter melon (BM) 30 on control	Half a teaspoon (2500 mg) of BM powder daily for 6 months (26 weeks)	Air-dried BM powder	47.5 ± 6.3 48.1 ± 5.9	NR	30 30	FBG, HbA1c
Mes (A) et al., 2025 [51]	Netherlands	Randomised, double-blind, controlled, cross-over trial	30 prediabetes	15 on BG and 15 on placebo	2.4 g (2400 mg) of BG as six capsules daily for 4 weeks	Freeze-dried BG fruit juice	67.5 ± 6.6 68.1 ± 4.8	29.1 ± 2.2 28.3 ± 3.2	10/5 11/4	HbA1c, FBG Insulin, HOMA-β, HOMA-IR
Mes (B) et al., 2025 [51]	Netherlands	Randomised, double-blind, controlled parallel trial	36 prediabetes	19 on BG and 17 on placebo	3.6 g (3600 mg) of BG as nine capsules daily for 12 weeks	Freeze-dried whole fruits	62 ± 8.1 64.5 ± 5.7	30.3 ± 5.0 30.7 ± 4.0	11/8 7/10	HbA1c, FBG FPI, HOMA-β, HOMA-IR
Guarneiri (A) et al., 2024 [44]	USA	Randomised, parallel, placebo-controlled	45 prediabetes	23 on BM and 22 on placebo)	300 mg of bitter melon (BM) for 12 weeks	Bitter melon extract capsules	55.9 ± 10.9 53.9 ± 9.43	31.6 ± 4.32 31.6 ± 4.19	5/18 10/12	HbA1c, Glucose, Insulin, HOMA-β
Guarneiri (B) et al., 2024 [44]	USA	Randomised, parallel, placebo-controlled	45 prediabetes	23 on BM and 22 on placebo	600 mg of BM for 12 weeks	bitter melon extract capsules	56.9 ± 11.7 53.9 ± 9.43	33.1 ± 4.55 31.6 ± 4.19	8/15 10/12	HbA1c, Glucose, Insulin, HOMA-β
Kim et al., 2022 [55]	Korea	Randomised placebo-controlled clinical	65 prediabetes	33 on BM and 32 on placebo	Two tablets of 2.4 g/2400 mg BM three times daily (7200 mg) for 12 weeks	Dry fruit extract	56.7 ± 11.3 53.6 ± 7.6	NR	10/23 16/16	HbA1c, FBG, Insulin, HOMA-IR, HOMA-β
Yang et al., 2022 [56]	Taiwan	Randomised, double-blind, placebo-controlled, parallel	40 T2D	20 on the extract and 20 on the placebo	600 mg/day (mcIRBP-19-BGE) for 3 months (13 weeks)	mcIRBP-19 containing extract	58.3 ± 12.7 58.6 ± 13.9	26.0 ± 4.2 26.3 ± 4.5	6/14 5/15	HbA1c FBG
Majeed (A) et al., 2021 [63]	India	prospective, randomised, double--blind, active-controlled clinical trial	29 prediabetes	17 on Glycacare II 12 on metformin	522.5 mg Glycacare II twice a day (1045 mg) for 120 days (17 weeks)	*Cinnamomum cassia, Momordica charantia, Pterocarpus marsupium, Gymnema sylvestre, Salacia reticulata,* *Eugenia jambolana* and *Piper nigrum*.	49.6 48	26.87 28.31	9/8 3/9	HbA1c
Majeed (B) et al., 2021 [63]	India	prospective, randomised, double--blind, active-controlled clinical trial	40 newly diagnosed T2D	24 ON Glycacare II 16 on metformin	522.5 mg Glycacare II twice a day (1045 mg) for 120 days (17 weeks)	*Cinnamomum cassia, Momordica charantia, Pterocarpus marsupium, Gymnema sylvestre, Salacia reticulata,* *Eugenia jambolana*, *Piper nigrum*.	51.3 52.9	25.02 25.57	17/7 4/12	HbA1c
Kim et al., 2020 [54]	Korea	Single-centre, randomised, double blind, placebo-controlled	90 T2D	62 on BM, 28 on placebo	2380 mg of BM per day for 12 weeks	Dry fruit extract	58.1 ± 6.9 60.3 ± 7.6	25.3 ± 3.8 26.6 ± 5.0	38/24 12/16	HbA1c, FBG, HOMA-IR, HOMA-β
Bumrungpert et al., 2020 [46]	Thailand	Randomise, double-blind, placebo-controlled trial	102 hyperglycemic	52 nutraceuticals 50 on placebo	2 nutraceuticals (500 mg) after breakfast and lunch Daily (1000 mg) for 12 weeks	Bitter melon fruit Fenugreek seed Cinnamon stem bark Alpha lipoic acid, Zinc Biotin, Chromium, Cholecalciferol	49.91 ± 12.20 50.83 ± 11.30	25.46 ± 4.88 25.52 ± 4.51	11/41 8/42	HbA1c, FBG
Hsu et al., 2020 [52]	Taiwan	Randomised controlled trial	142 DM	64 on BM and 78 in control	300 mg BMP twice (600 mg) for 3 months (13 weeks)	Powder from bitter melon	62.9 ± 12.1 59.3 ± 11.9	NR	NR	HbA1c, FBG, Insulin
Zhu et al., 2019 [64]	China	Double-blinded, randomised placebo control trial	88 T2D	43 on *M. charantia* 45 on placebo	1 tablet (500 mg) of GlucoVita supplements twice a day (1000 mg) for 12 weeks	*Momordica charantia*, plus Gymnema, Fenugreek, Indian tinospora, Kino tree, Bael tree, Neem, Cinnamon, and Cluster fig.	63.57 ± 8.11 63.03 ± 7.66	25.64 ± 3.42 25.63 ± 3.30	17/26 15/30	HbA1c, FBG
Cortez-Navarrete et al., 2018 [47]	Mexico	Randomised, double-blinded, placebo-controlled	24 T2D	12 on *M. charantia,* 12 on placebo	Two capsules orally of 500 mg M*. charantia* twice daily (1000 mg) for 12 weeks	Dried powder of the fruit pulp	50.1 ± 7.3 47.0 ± 7.4	29.1 ± 2.4 28.8 ± 3.9	5/7 3/9	HbA1c, FBG
Krawinkel et al., 2018 [48]	Tanzania	Randomised, single-blinded, placebo-controlled, crossover	52 prediabetes	28 on BM and 24 on placebo	2.5 g (2500 mg) of BG powder for eight weeks	Bitter gourd powder	48.2 ± 8.4 46.6 ± 9.1	29.1 ± 2.0 30.2 ± 2.5	23/27 23/27	HbA1c, FBG, FPI
Nakanekar et al., 2019 [61]	India	Double-blinded, placebo-controlled, randomised clinical trial.	114 prediabetes	57 on PDBT 57 on placebo	2 g (2000 mg) of PDBT for 6 months (26 weeks)	Aqueous extracts of the stem of Tinospora cordifolia, Miers, bark of Pterocarpus marsupium Roxburgh., leaves of Gymnema sylvestre R. Br., rhizome of Zingiber officinale Roscoe. and the fruit of Momordica charantia L.	51 (46e59) 45 (39e52)	27.5 (2.9) 29.1 (3.9)	19/38 27/30	HbA1c, FBG FPI, HOMA-IR, HOMA-β
Kumari (A) et al., 2018 [53]	India	Parallel randomised controlled trial	50 T2D	25 on *M. charantia* and 25 on oral anti-diabetic agents	1 g (1000 mg) of *M. charantia* tablets with oral anti-diabetic agents for 8 weeks	*Momordica charantia* tablets	40–60	26.3 ± 3.7 28.9 ± 5.4	NR	HbA1c, FBG, Insulin
Kumari (B) et al., 2018 [53]	India	Parallel, randomised controlled trial	50 T2D	25 on *M. charantia* plus oral anti diabetic agents, 25 on oral hypoglycaemic drugs	1.5 g (1500 mg) of *M. charantia* tablets plus oral anti diabetic agents for 8 weeks	*Momordica charantia* tablets	40–60	27.95 ± 3.2 28.9 ± 5.4	NR	HbA1c, FBG, Insulin
Amirthaveni (A) et al., 2018 [58]	India	Single-blinded, placebo-controlled, randomised, crossover	65 prediabetes	35 on MC and 30 on placebo	2.5 g (2500 mg) of BG powder for 8 weeks	Dried bitter gourd powder	30–50	27.64 ± 5.89 27.16 ± 4.57	18/27 35/10	HbA1c, FBG, Insulin
Amirthaveni (B) et al., 2018 [58]	India	Single-blinded, placebo-controlled, randomised, crossover	60 prediabetes	30 on MC 30 on placebo	2.5 g (2500 mg) of BG juice for 8 weeks	Dried bitter gourd juice	30–50	25.51 ± 3.77 25.41 ± 3.75	18/27 35/10	HbA1c, FBG, Insulin
Yu et al., 2018 [45]	China	Randomised, positive-controlled, open-label clinical trial	414 T2D	219 on *M. charantia* supplement (JTTZ) 199 on metformin	30 g (30000 mg) of JTTZ as granules 2 times daily (60000 mg) for 12 weeks	*M. charantia* plus Aloe vera, Coptis chinensis, Rhizoma Anemarrhena, red yeast rice, Salvia miltiorrhiza, Schisandra chinensis, and dried ginger	52.82 ± 9.01 52.90 ± 8.52	28.24 ± 3.31 28.01 ± 3.22	104/111 98/101	HbA1c, FBG, HOMA-IR, HOMA-β
Azam et al., 2016 [62]	Indonesia	Prospective, randomised, double-blind, comparative positive controlled, cross-over, clinical trial.	41 T2D	21 on extract 20 on glibenclamide	22 mg per day for a month (≈4 weeks)	*Pterocarpus indicus, Momordica charantia, Phaseolus vulgaris* and *Andrographis paniculata*.	49.81 ± 7.41 49.75 ± 6.97	26.44 ± 10.45 26.59 ± 4.48	4/17 5/15	HbA1c FBG
Suthar (A) et al., 2016 [66]	India	Open-label, randomised, active-controlled, phase III study	111 T2D	75 on *M. charantia* 36 on metformin	400 mg of *M. charantia* capsule twice a day (800 mg) for 15 weeks	Unripe green fruits of *M. charantia* plant	49.43 ± 9.26 47.70 ± 9.11	26.96 ± 4.46 27.62 ± 3.49	46/37 23/17	FBG HbA1c
Suthar (B) et al., 2016 [63]	India	Open label, randomised, active controlled, Phase II trial	79 T2D	64 on the drug and 17 on the placebo	400 mg of *M. charantia* capsule three times (1200 mg) for 90 days (≈13 weeks)	Dry fruit juice powder of *Momordica charantia*	41.33 ± 7.59 41.31 ± 6.88	NR	39/23 10/7	HbA1c, FBG
Rahman (A) et al., 2015 [49]	Pakistan	Randomised, double blind, parallel-group trial	59 T2D	30 on bitter melon 29 on glibenclamide	2 g (2000 mg) of BM, glibenclamide 2.5 mg for 10 weeks	Bitter melon powder in capsules	51.90 ± 10.50 52.20 ± 8.70	25.2 ± 1.70 25 ± 2.20	21/9 18/11	FBG
Rahman (B) et al., 2015 [49]	Pakistan	Randomised, double blind, parallel group trial	60 T2D	31 on bitter melon, 29 on glibenclamide	4 g (4000 mg) of BM, glibenclamide 2.5 mg for 10 weeks	Bitter melon powder in capsules	52 ± 11.40 52.20 ± 8.70	25.7 ± 2.40 25 ± 2.20	20/11 18/11	FBG
Trakoon-osot et al., 2013 [59]	Thailand	Two-arm, parallel, randomised, placebo-controlled trial	38 T2D	19 on MC and 19 on placebo	6 g (6000 mg) of MC dried-fruit pulp for 16 weeks	Dried pulp	57.2 ± 8.8 58.7 ± 7.0	25.04 ± 3.69 26.37 ± 6.04	3/16 8/11	HbA1c, FBG
Zänker (A) et al., 2012 [57]	Germany	Randomised, double-blinded, and Placebo-controlled	97 T2D	30 *M. charantia* and 32 placebos	1 capsule (0.5 g/500 mg) BM powder twice daily (1000 mg) for four months (17 weeks)	Dried fruit	63.5 ± 7.76 61.5 ± 7.26	30.6 ± 5.97 28.4 ± 4.51	21/9 23/9	HbA1c
Zänker (B) et al., 2012 [57]	Germany	Randomised, double-blinded, and placebo-controlled	67 T2D	35 *M. charantia* + chromium + zinc and 32 on placebo	0.5 g (500 mg) of BM twice (1000 mg) plus 50 μg Chromium, 5 mg of zinc for 4 months (17 weeks)	Dried fruit	62.8 ± 8.53 61.5 ± 7.26	28.9 ± 4.37 28.4 ± 4.51	26/9 23/9	HbA1c
Fuangchan (A) et al., 2011 [50]	Thailand	Randomised, double-blind, active-control trial	66 T2D	33 on BM 33 on metformin	500 mg of BM and Metformin 1000 mg/day for 4 weeks	Dried fruit pulps	52.2 ± 8.3 52.5 ± 9.2	25.0 ± 3.4 24.4 ± 3.1	8/25 9/24	FBG
Fuangchan (B) et al., 2011 [50]	Thailand	Randomised, double-blind, active-control trial	65 T2D	32 on BM 33 on metformin	1000 mg of BM and Metformin 1000 mg for 4 weeks	Dried fruit pulps	50.6 ± 10.7 52.5 ± 9.2	25.6 ± 3.9 24.4 ± 3.1	6/26 9/24	FBG
Fuangchan (C) et al., 2011 [50]	Thailand	Randomised, double-blind, active-control trial	64 T2D	31 on BM 33 on metformin	2000 mg of BM and Metformin 1000 mg for 4 weeks	Dried fruit pulps	52.0 ± 9.1 52.5 ± 9.2	25.4 ± 3.0 24.4 ± 3.1	11/20 9/24	FBG
Dans et al., 2007 [28]	Philippines	Randomised, double-blind, placebo-controlled trial	40 T2D	19 in *M. Charania* and 20 on placebo	Two capsules of 3 g (3000 mg) *M. charantia* three times daily (18000 mg) of 3 months (13 weeks)	Extract from fruits and seeds	58.70 ± 9.81 59.76 ± 10.04	26.37 ± 4.75 26.00 ± 3.94	7/16 8/12	HbA1c, FBG
John et al., 2003 [29]	India	Randomised controlled clinical trial	50 T2D	26 on BG and 24 on placebo	2 tablets of 1 g (1000 mg) of BG three times a day (3000 mg) for 3 weeks	Dried fruit	52.25 ± 8.77 54.25 ± 9.839	NR	7/19 9/15	FBG

T2D: type 2 diabetes; FBG: fasting blood glucose; HOMA-IR: homeostatic model assessment of insulin resistance; HOMA-β: homeostatic model assessment for beta cell function; BG: bitter gourd; BM: bitter melon; RCT: randomised controlled trial; HbA1c: glycated haemoglobin; NR: not reported; BMI: body mass index.

### 3.3 Risk of bias, quality assessments and certainty of evidence

The overall individual trial ROB and quality are presented in Appendix B, Fig. 1A and B. Across all 34 studies, only three [60,65,66] were rated as high risk of bias, implying they have low quality due to missing information about the process of randomisation and deviation from the intended intervention. Thirteen trials [29,49,[51], [52], [53],[55], [56], [57], [58], [59], [60], [61],^63^] showed some concerns for this domain, as they did not provide a clear method of how randomisation was done. For bias in the selection of reported results, 13 studies [29,49,52,53,55,57,59,60,[62], [63], [64], [65], [66]] did not provide information about the protocol registration, which makes it difficult to determine whether researchers have reported results as planned or not. Of these studies, seven trials [28,44,46,47,50,54] were classified as good, 16 trials [29,45,48,49,[51], [52], [53],[55], [56], [57], [58], [59],[61], [62], [63], [64]] as moderate and three trials [60,65,66] as poor quality. The certainty of evidence was GRADED and presented as a summary of findings table in, Appendix B Table 2. The evidence analysed for FBG, insulin, and HOMA-IR was downgraded by two levels due to bias associated with two domains: risk of bias and indirectness. Hence, the evidence was classified as of low quality. For HbA1c, the risk of bias, indirectness, and publication bias were noted; therefore, the evidence was classified as very low. For HOMA-β, evidence was also downgraded by two points due to bias associated with the risk of bias and imprecision, as indicated by narrow confidence intervals.

### 3.4 Effect of *M. charantia* on fasting blood glucose and HbA1c

**Fig. 2:**
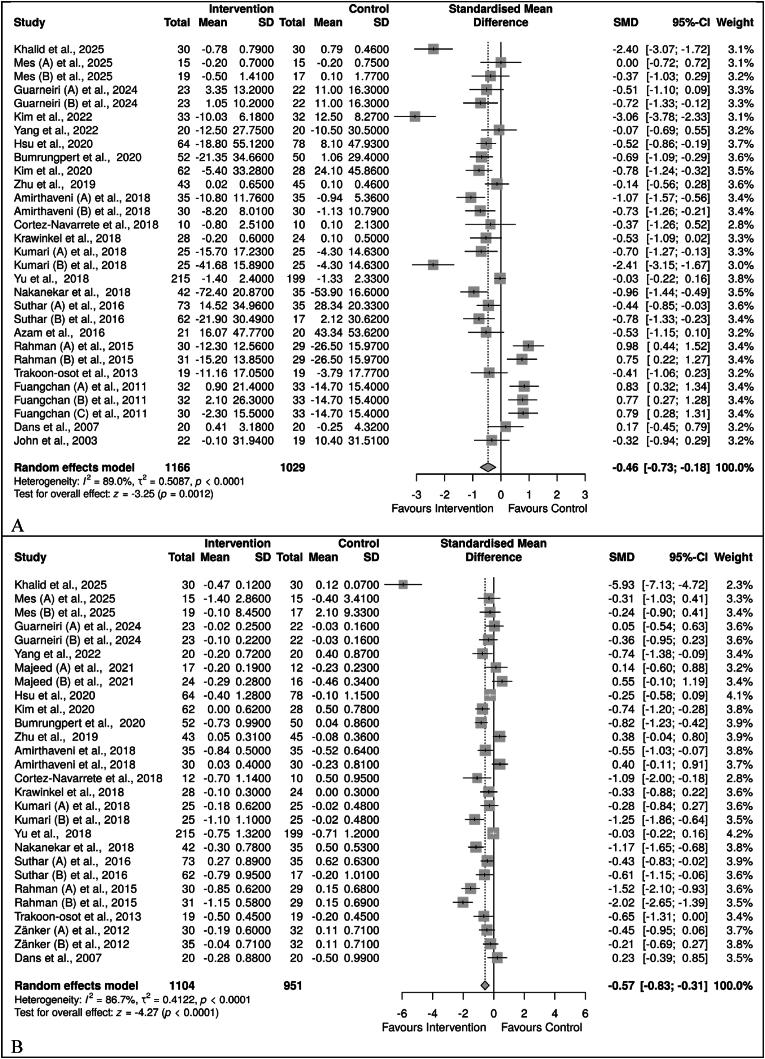
Effect of *M. charantia* on FBG in prediabetes and type 2 diabetes mellitus. A. FBG, B. HbA1c.

The data from 23 trials with 31 substudies [28,29,[44], [45], [46], [47], [48], [49], [50], [51], [52], [53], [54], [55], [56],[58], [59], [60], [61], [62],[64], [65], [66]] were analysed for the effect of *M. charantia* on FBG in prediabetics and T2D. Due to high heterogeneity (*I*^2^ = 89 %), a random effect model meta-analysis was employed. The results showed a reduction in FBG following *M. charantia* compared to the control group, with an SMD of −0.46, a 95 % CI of (−0.78, −0.14), and a p-value of 0.0043 (Fig. 2A). Only 20 trials with 27 substudies [28,[44], [45], [46], [47], [48], [49], [50], [51], [52], [53], [54],[56], [57], [58], [59], [60], [61],[63], [64], [65], [66]] were analysed for HbA1c in prediabetes and T2D. Due to high heterogeneity (*I*^2^ = 87 %), a random-effect model meta-analysis was conducted. The evidence showed that *M. charantia,* compared to control, significantly reduced HbA1c, SMD = −0.57, 95 %CI (−0,83, −0.31), *p* < 0.0001 (Fig. 2B)

### 3.5 Effect of *M. charantia* on insulin, the homeostatic model assessment of insulin resistance and the homeostatic model assessment of beta function

**Fig. 3:**
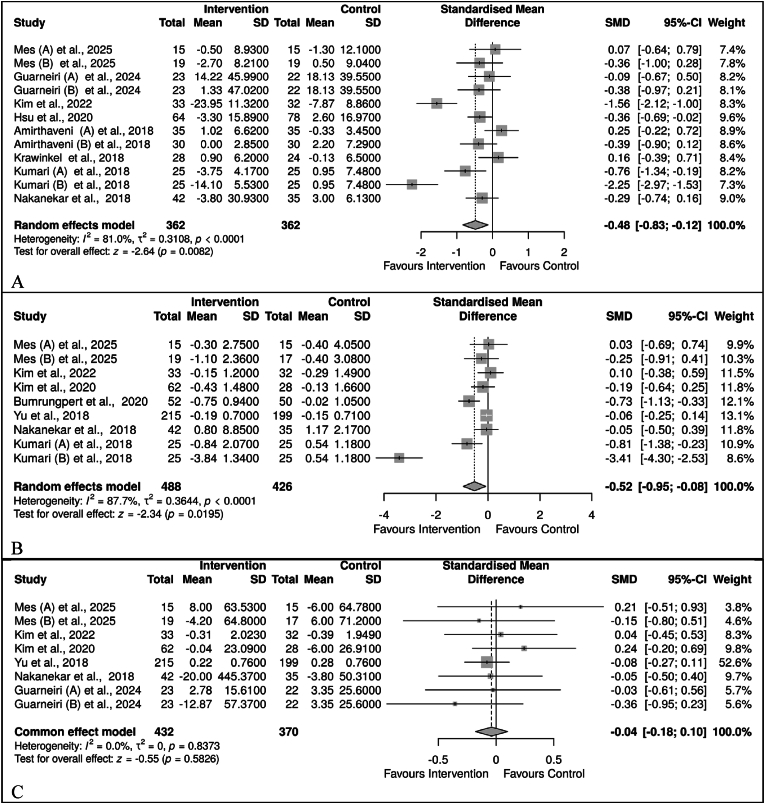
Effect of *M. charantia* on insulin resistance in prediabetes and T2D. A. Insulin, B. HOMA-IR, C. HOMA-β

The data from 8 trials with 12 substudies [44,48,[51], [52], [53],^55^,^58^,^61^] on insulin were analysed. Due to high heterogeneity (*I*^2^ = 81 %), a random-effects model meta-analysis was employed. The overall effect showed a reduced insulin level in *M. charantia* compared to the control, SMD = −0.48, 95 %CI (−0.83, −0.12), *p* = 0.0082 (Fig. 3B). Additionally, the five trials [45,46,51,54,55,61] analysed for HOMA-IR, showed a significant reduction in its level following supplementation with *M. charantia*; SMD = −0.52, 95 % CI (−0.95, −0.08), *p* = 0.0195. This was associated with a high level of heterogeneity (*I*^2^ = 88 %). Six trials [44,45,51,54,55,61] were analysed for HOMA-β; however, no significant effect of *M. charantia* on HOMA-β, SMD = −0.04, 95 %CI (−0.18, 0.10), *p* = 0.5826. Interestingly, there was no evidence of heterogeneity (*I*^2^ = 0 %) (Fig. 3C).

### 3.6 Subgroup analysis

The presented evidence showed significant heterogeneity across all parameters. This prompted a rigorous subgroup analysis based on dose of *M. charantia*, duration of intervention (>12 or ≤ 12 weeks), continent of publication, and ROB as presented in, Appendix B Table 3. Regarding FBG, trials that used a high dose of *M. charantia* (three capsules of 30 g *M. charantia* taken three times a day or 30 g of *M. charantia* granule taken twice a day) [28,45]. Our subgroup analysis revealed that high doses of *M. charantia* and trials conducted in Europe and America were significant contributors to the observed heterogeneity for FBG and HbA1c. Similarly, for insulin, low-dose and intervention periods exceeding 12 weeks, including trials published in Europe and America, as well as studies with a low risk of bias, were found to contribute to heterogeneity. For HOMA-IR, trials that used low doses and those published in Europe equally contributed to high heterogeneity.

### 3.7 Sensitivity test

For FBG, excluding the study by Kim et al. (2022) resulted in a 20 % decrease in effect size, SMD = −0.37 (−0.62, −0.12), *p* = 0.0038, *I*^2^ = 86.5 % [55]. When a trial by Khalid et al. (2025) was excluded from analysis of HbA1c due to poor quality, the resulting estimates showed a 22 % decrease in effect size, SMD = −0.44 (−0.65, −0.23), *p* < 0.0001, *I*^2^ = 78.6 % [65]. An exclusion of Kumari (B) et al. (2018) due to the use of *M. charantia* alongside other oral anti-diabetic agents led to a 29 % decrease in effect size on insulin with an effect size, SMD = −0.34 (−0.62, −0.05), *p* = 0.0194, *I*^2^ = 68.5 % [53]. Similarly, the exclusion of Kumari (B) et al. (2018) changed the effect size by at least 54 % (0.24 (−0.47, −0.00), *p* = 0.0457, *I*^2^ = 53.9 % for HOMA-IR [53]. On the other hand, the exclusion of the trial by Yu et al. (2018) due to its use of polyherbal extracts and large sample size demonstrated a substantial change, with results showing no difference between the groups, which is a 100 % increase from the initial effect size [SMD = 0.00, (−0.20 to 0.20, p = 0.964, *I*^2^ = 0 %] for HOMA-β.

### 3.8 Publication bias

**Fig. 4:**
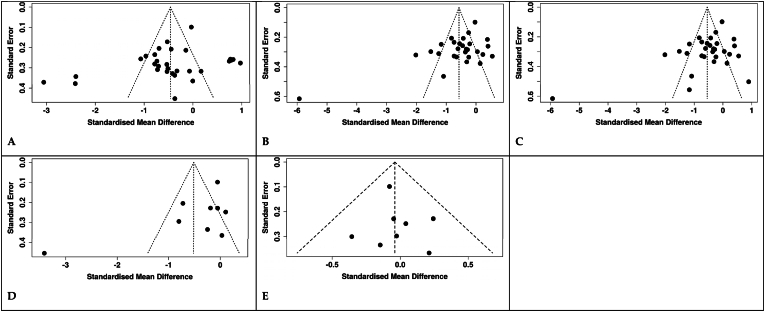
Visual inspection of funnel plot to assess publication bias, A. FBG, B. HbA1c, C. Insulin, D. HOMA-IR, E. HOMA-.

For FBG, visual inspection of the funnel plot showed no evidence of publication bias (Fig. 4A). This was supported by an Egger's test (*p* = 0.126). For HbA1c, we noted evidence of publication bias through visual inspection of the funnel plot (Fig. 4B). This was in line with the result of the Eggers regression test (*p* = 0.0016). Furthermore, the application of the trim and fill test showed a 65 % decrease in the pooled effect size (Appendix B, Fig. 2). This observation suggests that the initial effect size might have been overestimated due to bias, making it highly likely that this significantly impacted the overall findings of the meta-analysis on this marker. For both insulin and HOMA-IR, inspection of funnel plots showed no evidence of potential publication bias (Fig. 4C and D). These findings were further supported by Egger's *p*-values of 0.345 and 0.140, respectively. Similarly, no evidence of publication bias was observed visually (Fig. 4E and statistically (*p* = 0.641).

## 4 Discussion

Prediabetes and T2D are marked by chronic hyperglycaemia, characterised by elevated levels of FGB, HbA1c, insulin and HOMA-IR, along with impaired β-cell function [67,68]. An increase in these parameters in prediabetes and T2D patients predisposes them to the development of CVD and related mortality [69]. In this study, *M. charantia* led to a significant reduction in FBG, HbA1c, insulin, and HOMA, IR, suggesting it may improve insulin sensitivity in patients living with prediabetes and type 2 diabetes. However, the effect on β-cell function was not significant, indicating that pancreatic β-cell function was preserved. Altogether, these findings suggest that *M. charantia* may improve glycaemia by enhancing insulin sensitivity rather than stimulating β-cell insulin secretion in individuals with prediabetes and type 2 diabetes.

Several trials support the reduction in FBG following *M. charantia,* as supported by previous researchers [44,[52], [53], [54], [55],^58^,^60^,^66^]. The same trends were observed on HbA1c [49,53,54,65] and insulin [44,53,55]. This study supports the findings from another meta-analysis, which showed the potential effect of *M. charantia* on FBG and HbA1c [30], above that, it revealed a potential effect on insulin resistance, as demonstrated by reduced insulin levels alongside HOMA-IR, which had not been previously explored through quantitative analysis.

In contrast to our findings, a previous meta-analysis showed no effect on FBG and HbA1c [70]. It is worth noting that this previous meta-analysis consisted of only three trials, which limits its statistical power. Likewise, Laczkó-Zöld and the team also showed no effect of *M. charantia* on FBG and HbA1c [30]. Among other factors that might have contributed to the lack of effect in the latter study, the inclusion criteria could be a factor. The inclusion of obesity, diabetes, and healthy individuals likely contributed to the variation in results.

Although our findings suggest some potential benefits of *M. charantia* as an intervention in this study, it is essential to understand how *M. charantia* regulates hyperglycaemia in prediabetes and type 2 diabetes. The evidence from rodent models and in vivo studies suggests that the mechanisms through which *M. chatantia* exerts its antihyperglycaemic properties involve various pathways. For instance, evidence from prediabetes and T2D rodent models suggests this activity may be mediated by *M. charantia's* high content of alkaloids and flavonoids [[71], [72], [73]]. These phytochemicals promote the antihyperglycaemic activities of *M. charantia* through the activation of glucose transporters (GLUT) in skeletal muscle and fat cells, thereby enhancing glucose absorption and reducing blood glucose levels [74]. Moreover, the high content of alkaloids in *M. charantia* activates hepatic enzymes, such as glycogen synthase (GS), and inhibits glucose-6-phosphatase, thereby promoting the conversion of excess glucose into glycogen for storage [75,76]. Recent evidence demonstrated an increased hepatic and muscle glycogen level after treatment with *M. charantia* polysaccharide in diabetic C57BL/6 mice [26]. This activity can further maintain glucose homeostasis by increasing glycogen synthesis.

**Fig. 5:**
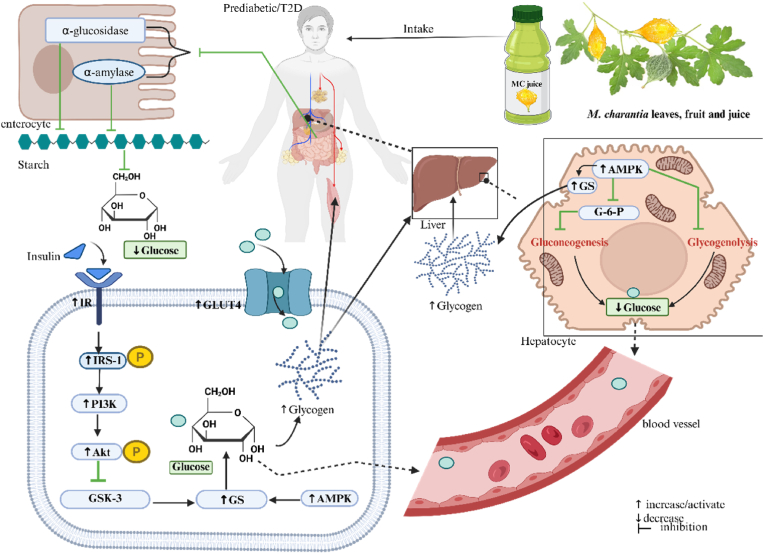
Proposed mechanism by which *M. charantia* lowers blood glucose [26,77]. GLUT-4: glucose transporter 4, IR: insulin resistance, IRS-1: insulin resistance substrate 1, PI3K: phosphoinositide 3-kinase, Atk: protein kinase β, GSK-3K: glycogen synthase kinase 3, GS: glycogen synthase, AMPK: adenosine monophosphate protein kinase, G-6-P: glucose-6-phosphatase. Biorender was used to create the figure.

In addition*, M. charantia* modulates the adenosine monophosphate-activated protein kinase (AMPK) and peroxisome proliferator-activated receptor alpha (PPAR-α) signalling pathways [26,77]. An active AMPK promotes glycogen synthase, an enzyme that facilitates glycogen synthesis, thereby limiting blood glucose levels. Notably, *M. charantia* is involved in gluconeogenesis by inhibiting glucose-6-phosphatase, thereby reducing blood glucose levels (Fig. 5). In diabetic mice, *M. charantia* was associated with the activation of the insulin receptor substrate 1 (IRS-1) and the upregulation of the phosphoinositide 3-kinase (PI3K) pathway [26]. The role of *M. charantia* on these pathways collectively increases glucose uptake by promoting the translocation of GLUT-4 to the plasma membrane in muscle and adipose tissue, thus reducing blood glucose and improving hyperglycaemia [73].

Another study demonstrated that *M. charantia* inhibits α-amylase and α-glucosidase, enzymes involved in carbohydrate breakdown, leading to reduced glucose levels [71,78]. It is worth noting that these inhibitions seem to be concentration-dependent; the higher the concentration, the stronger the inhibition [71]. Indeed, the evidence presented in this study suggests the potential of *M. charantia* to reduce hyperglycaemia based on reduced FBG, insulin, HbA1c and HOMA-IR in individuals living with prediabetes and T2D. Despite the positive impact on the above markers of hyperglycaemia, no significant effect was noted on HOMA-β, which suggests that *M. charantia* may ameliorate hyperglycaemia by improving insulin sensitivity rather than overworking the β-cells to increase insulin secretion. Therefore, the clinical evidence presented in this study suggests that *M. charantia* may have antihyperglycaemic potential, especially in patients with prediabetes and T2D.

To the best of our knowledge, this is the first study to analyse the high level of evidence and report the potential effect of *M. charantia* on FBG, HbA1c, insulin, and HOMA-IR. It is also registered on PROSPERO to allow transparency in research. Different databases, including PubMed/Medline, Scopus, and Web of Science, as well as manual screening, were utilised comprehensively to search for clinical evidence. A reasonable number of trials (26) were analysed, which improved the statistical power when compared to previous studies. The number of studies analysed was distributed across different countries (including Pakistan, the Netherlands, the USA, Thailand, Korea, Taiwan, China, Mexico, Tanzania, India, Germany, and the Philippines), which may provide a slightly representative picture of the global effect of *M. charantia* on these conditions. However, it is essential to note that these trials employed different dosages (from low to the highest) and had varying intervention periods, which could have introduced heterogeneity. Due to evidence of publication bias on HbA1c, the trim and fill analysis also suggested that the initial effect size might have been overestimated, as it was reduced by 65 %. While statistical heterogeneity was observed, the subgroup analysis revealed that this was due to trials using high doses of FBG and HbA1c, or low doses of insulin and HOMA-IR. Publications from America and Europe also contributed to the statistical heterogeneity. Hence, due to a lack of information about the formation method of *M. charantia*, subgroup analysis on this was not performed.

## 5 Conclusions

The evidence gathered in this study showed that *M. charantia* in individuals living with prediabetes and T2D may significantly reduce FBG, HbA1c, insulin and HOMA-IR levels without affecting HOMA-β. The results taken altogether suggest that *M. charantia* may reduce hyperglycaemia by improving insulin sensitivity without directly affecting the pancreatic β-cell function. Despite these potential findings, we recommend that future trials focus on determining the optimal dose and duration of intervention that can ameliorate hyperglycaemia in those living with prediabetes and T2D, especially since our analyses did not show any effect on HOMA-β, one of the markers of insulin resistance.

## CRediT authorship contribution statement

**Sphesihle A.L. Mkhize:** Writing – review & editing, Writing – original draft, Methodology, Investigation, Formal analysis, Data curation. **Wendy N. Phoswa:** Writing – review & editing, Visualization, Validation, Supervision, Methodology, Investigation, Data curation. **Phikelelani S. Ngubane:** Writing – review & editing, Visualization, Validation, Data curation. **Kabelo Mokgalaboni:** Writing – review & editing, Writing – original draft, Visualization, Validation, Supervision, Software, Resources, Project administration, Methodology, Investigation, Funding acquisition, Formal analysis, Data curation, Conceptualization.

## Data and resource availability

All data analysed in this study are available from the corresponding author upon reasonable request.

## Generative AI disclosure

AI and AI-assisted technologies were not used in the writing process of the manuscript.

## Funding

This research did not receive any specific grant from funding agencies in the public, commercial, or not-for-profit sectors.

## Conflict of interests

The authors declare that they have no known competing financial interests or personal relationships that could have appeared to influence the work reported in this paper.
