# Supplementary material for: Efficacy of Momordica charantia in glycaemic control and insulin resistance among patients with prediabetes and type 2 diabetes. A GRADE-adherent meta-analysis of randomised controlled trials
Source: Metabol Open. 2025 Oct 24;28:100407. doi: 10.1016/j.metop.2025.100407 (PMC12630335; doi:10.1016/j.metop.2025.100407)
Supplement: Multimedia component 1 [file mmc1.docx]

**Appendix B**

**Table 1:** search strategy adapted to the databases.

| **Database** | **Exact search and restrictions** | **Records** |
| --- | --- | --- |
| PubMed | (((Diabetes mellitus [MeSH Terms]) OR (Prediabetes [MeSH Terms])) AND (Momordica charantia [MeSH Terms])) OR (Bitter melon [MeSH Terms]) Filters: Randomized Controlled Trial | 10 |
| Scopus | (TITLE-ABS-KEY (Momordica charantia) OR TITLE-ABS-KEY (bitter melon) AND TITLE-ABS-KEY (diabetes mellitus) OR TITLE-ABS-KEY (prediabetes) AND TITLE-ABS-KEY (randomized controlled trial)) AND (LIMIT-TO (DOCTYPE, "ar")) AND (LIMIT-TO (LANGUAGE, "English")) AND (LIMIT-TO (SRCTYPE , "j")) | 39 |
| Web of Science | ((((TS=(Diabetes mellitus)) OR TS=(prediabetes)) AND TS=(Momordica Charantia)) OR TS=(Bitter melon)) AND TS=(randomized controlled trials) | 45 |

| **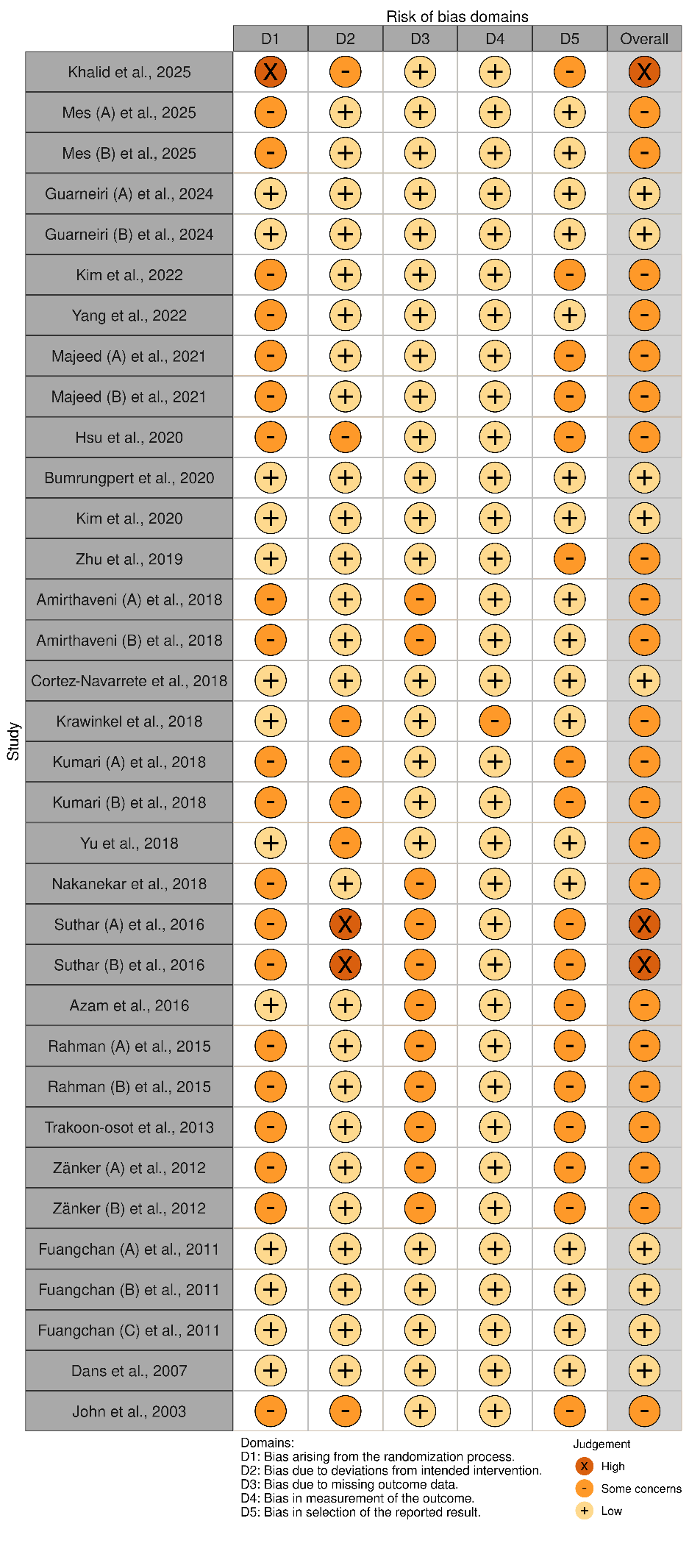** | **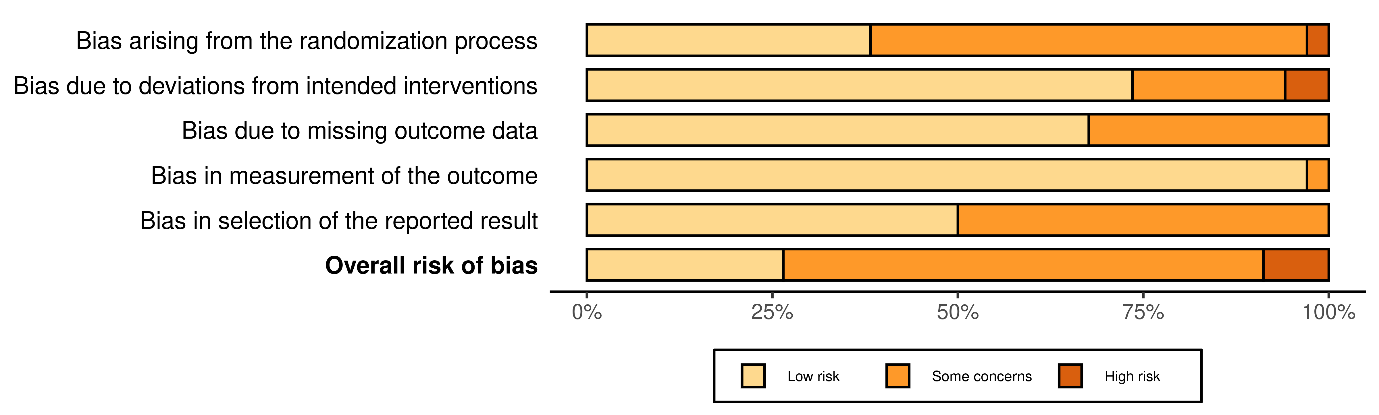** |
| --- | --- |
|  |  |

**Figure 1:** Risk of bias, quality assessment. A. Individual trial ROB plot, B. ROB summarised the plot

**Table 2:** Summary of findings table (SOF) showing certainty of presented evidence

| **Certainty assessment** | | | | | | | **Number of participants** | | **Effect size** | **Heterogeneity** | **Certainty of evidence** |
| --- | --- | --- | --- | --- | --- | --- | --- | --- | --- | --- | --- |
| **Outcome** | **Number of studies** | **Risk of bias** | **Inconsistency** | **Indirectness** | **Imprecision** | **Other considerations** | **Intervention** | **Control** | **SMD (95% CI)** | ***I*^2^ ( %)** |  |
| FBG | 30 | Serious^a^ | Serious^b^ | Not serious | Not serious | None | 1166 | 1029 | -0.46 (-0.73,- 0.18* | 89 | ⨁⨁◯◯  Low^a,b^ |
| HbA1c | 28 | Serious^a^ | Serious^b^ | Not serious | Not serious | Publication bias strongly suspected^c^ | 104 | 951 | -0.57 (-0.83, -0.31* | 87 | ⨁◯◯◯ Very low^a,b,c^ |
| Insulin | 12 | Serious^d^ | Serious | Not serious | Not serious | None | 362 | 362 | = -0.48 (-0.83, -0.12* | 81 | ⨁⨁◯◯  Low^b,d^ |
| HOMA-IR | 9 | Serious^d^ | Serious^b^ | Not serious | Not serious | None | 488 | 426 | -0.52 (-0.95, -0.08)* | 88 | ⨁⨁◯◯  Low^b,d^ |
| HOMA-β | 8 | Serious^d^ | Not serious | Not serious | Serious^e^ | None | 432 | 370 | -0.04, 95%CI (-0.18, 0.10) | 0 | ⨁⨁◯◯  Low^d,e^ |

a. Downgraded by one as the majority of domains were judged as having “some concerns,” some “high risk,” b. High I square test value. c. Funnel plot and Egger's test (*p* < 0.05 indicates potential bias. d. Several trials have “some concerns”. e. The primary confidence intervals crosses the null (upper bound +0.10), indicating imprecision.

Table 3: Results of subgroup analysis for outcomes showing heterogeneity

| **Outcome** | **Subgroups** | **Class** | **Studies** | **Effect size** | ***I*^2^** |
| --- | --- | --- | --- | --- | --- |
| FBG | Dose | Very low | 1 | -0.46 (-0.73, -0.18) | N/A |
|  |  | Low | 11 | -0.23 (-0.56, 0.10) | 78.8 |
|  |  | Standard | 16 | -0.69 (-1.2, -0.18) | 92.2 |
|  |  | High | 2 | -0.01 (-0.20, 0.17) | 0 |
|  |  |  |  |  |  |
| HbA1c | Dose | Very low | 0 |  |  |
|  |  | Low | 11 | -0.34 (-0.57, -0.11) | 57.3 |
|  |  | Standard | 15 | -0.87 (-1.37, -0.38) | 90.4 |
|  |  | High | 2 | -0.01 (-0.19, 0.18) | 0 |
|  |  |  |  |  |  |
| Insulin | Dose | Very low | 0 |  |  |
|  |  | Low | 4 | -0.38 (-0.62, -0.15) | 0 |
|  |  | Standard | 8 | -0.53 (-1.09, 0.03) | 87.3 |
|  |  | High | 0 |  |  |
|  |  |  |  |  |  |
| HOMA-IR | Dose | Very low |  |  |  |
|  |  | Low | 2 | -0.75 (-1.08, -0.42) | 0 |
|  |  | Standard | 6 | -0.57 (-1.32, 0.18) | 90.4 |
|  |  | High | 1 | -0.06 (-0.25, 0.14) | NA |
|  |  |  |  |  |  |
| FBG | Duration (weeks) | ≤ 12 | 22 | -0.38 (-0.72, -0.04) | 89.9 |
|  |  | > 12 | 8 | -0.67 (-1.10, -0.23) | 82.4 |
|  |  |  |  |  |  |
| HbA1c | Duration (weeks) | ≤ 12 | 16 | -0.52 (-0.83, -0.21) | 84.1 |
|  |  | > 12 | 12 | -0.67 (-1.16, -0.18) | 89.8 |
|  |  |  |  |  |  |
| Insulin | Duration (weeks) | ≤ 12 | 10 | -0.52 (-0.98, -0.05) | 84.3 |
|  |  | > 12 | 2 | -0.33 (-0.60, -0.06) | 0 |
|  |  |  |  |  |  |
| HOMA-IR | Duration (weeks) | ≤ 12 | 8 | -0.59 (-1.08, -0.09) | 89.1 |
|  |  | > 12 | 1 | -0.05 (-0.50, 0.39) | NA |
|  |  |  |  |  |  |
| FBG | Continent | Africa | 1 | -0.53 (-1.09, 0.02) | NA |
|  |  | Asia | 24 | -0.47 (-0.79, -0.14) | 91.1 |
|  |  | Europe | 2 | -0.20 (-0.68, 0.29) | 0 |
|  |  | America | 3 | -0.57 (-0.95, -0.19) | 0 |
|  |  |  |  |  |  |
| HbA1c | Continent | Africa | 1 | -0.33 (-0.88, 0.22) | NA |
|  |  | Asia | 20 | -0.67 (-1.02, -0.33) | 90.4 |
|  |  | Europe | 4 | -0.31 (-0.59, -0.02) | 0 |
|  |  | America | 3 | -0.38 (-0.95, 0.19) | 53.2 |
|  |  |  |  |  |  |
| Insulin | Continent | Africa | 1 | 0.16 (-0.39, 0.71) | NA |
|  |  | Asia | 7 | -0.73 (-1.26, -0.20) | 87.7 |
|  |  | Europe | 2 | -0.17 (-0.65, 0.31) | 0 |
|  |  | America | 2 | -0.23 (-0.65, 0.16) | 0 |
|  |  |  |  |  |  |
| HOMA-IR | Continent | Africa | 0 |  |  |
|  |  | Asia | 7 | -0.63 (-1.15, -0.11) | 90.7 |
|  |  | Europe | 2 | -0.12 (-0.61, 0.36) | 0 |
|  |  | America | 0 |  |  |
|  |  |  |  |  |  |
| FBG | ROB | High risk | 3 | -1.18 (-2.25, -0.11) | 91.7 |
|  |  | Low risk | 9 | -0.05 (-0.54, 0.44) | 86.5 |
|  |  | Some concerns | 18 | -0.54 (-0.89, -0.19) | 89.1 |
|  |  |  |  |  |  |
| HbA1c | ROB | High risk | 3 | -2.22 (-4.38, -0.06) | 97.3 |
|  |  | Low risk | 6 | -0.45 (-0.83, -0.06) | 64.2 |
|  |  | Some concerns | 19 | -0.44 (0.71, -0.17) | 82.7 |
|  |  |  |  |  |  |
| Insulin | ROB | High risk | 0 |  |  |
|  |  | Low risk | 2 | -0.23 (-0.65, 0.18) | 0 |
|  |  | Some concerns | 10 | -0.53 (-0.95, -0.11) | 84.1 |
|  |  |  |  |  |  |
| HOMA-IR | ROB | High risk | 0 |  |  |
|  |  | Low risk | 2 | -0.47 (-0.99, 0.05) | 67.1 |
|  |  | Some concerns | 7 | -0.56 (-1.13, 0.02) | 89.8 |

FBG: fasting blood glucose; HOMA-IR: homeostatic model assessment of insulin resistance; HOMA-β: homeostatic model assessment for beta cell function; HbA1c: glycated haemoglobin; NA: not applicable; ROB: risk of bias.

**
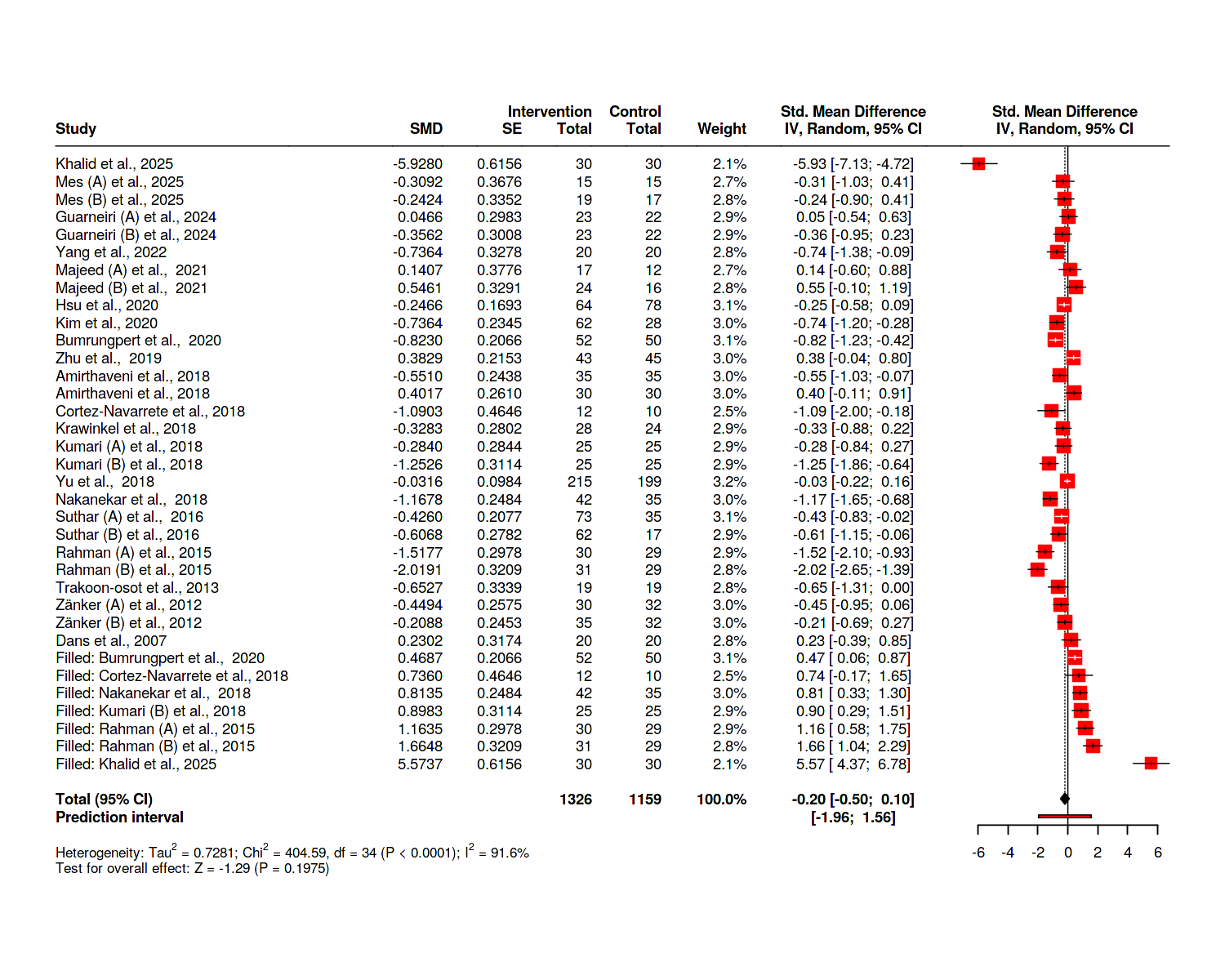
**

**Figure 2:** A trim and fill meta-analysis of findings on HbA1c
